# Computer-supported feedback message tailoring: theory-informed adaptation of clinical audit and feedback for learning and behavior change

**DOI:** 10.1186/s13012-014-0203-z

**Published:** 2015-01-21

**Authors:** Zach Landis-Lewis, Jamie C Brehaut, Harry Hochheiser, Gerald P Douglas, Rebecca S Jacobson

**Affiliations:** Center for Health Informatics for the Underserved, Department of Biomedical Informatics, University of Pittsburgh School of Medicine, Pittsburgh, PA USA; Ottawa Hospital Research Institute, Clinical Epidemiology Program, The Ottawa Hospital, Ottawa, ON Canada; School of Epidemiology, Public Health and Preventive Medicine, University of Ottawa, Ottawa, ON Canada; Intelligent Systems Program, University of Pittsburgh, Pittsburgh, PA USA

## Abstract

**Background:**

Evidence shows that clinical audit and feedback can significantly improve compliance with desired practice, but it is unclear when and how it is effective. Audit and feedback is likely to be more effective when feedback messages can influence barriers to behavior change, but barriers to change differ across individual health-care providers, stemming from differences in providers’ individual characteristics.

**Discussion:**

The purpose of this article is to invite debate and direct research attention towards a novel audit and feedback component that could enable interventions to adapt to barriers to behavior change for individual health-care providers: computer-supported tailoring of feedback messages. We argue that, by leveraging available clinical data, theory-informed knowledge about behavior change, and the knowledge of clinical supervisors or peers who deliver feedback messages, a software application that supports feedback message tailoring could improve feedback message relevance for barriers to behavior change, thereby increasing the effectiveness of audit and feedback interventions. We describe a prototype system that supports the provision of tailored feedback messages by generating a menu of graphical and textual messages with associated descriptions of targeted barriers to behavior change. Supervisors could use the menu to select messages based on their awareness of each feedback recipient’s specific barriers to behavior change. We anticipate that such a system, if designed appropriately, could guide supervisors towards giving more effective feedback for health-care providers.

**Summary:**

A foundation of evidence and knowledge in related health research domains supports the development of feedback message tailoring systems for clinical audit and feedback. Creating and evaluating computer-supported feedback tailoring tools is a promising approach to improving the effectiveness of clinical audit and feedback.

**Electronic supplementary material:**

The online version of this article (doi:10.1186/s13012-014-0203-z) contains supplementary material, which is available to authorized users.

## Background

Audit and feedback (AF) is defined as the provision of clinical performance summaries to health-care providers, teams, and organizations [1]. AF includes heterogeneous approaches used within multi-faceted interventions to change behavior for health-care quality improvement. Evidence from the most recent Cochrane review, including 140 clinical trials, shows that AF can significantly improve compliance with desired practice, but that it is unclear which approaches, under which circumstances, will work [2]. Given the limited insights produced by AF trials to date, AF researchers have called for a shift towards comparative effectiveness studies, evaluating how and when AF components will work, rather than its overall effectiveness [3].

AF is likely to be more effective when its components influence barriers to behavior change [4,5]. However, barriers to behavior change differ across health-care providers, stemming from differences in providers’ training, knowledge, work experience, personality, and other individual characteristics [6]. Furthermore, barriers may be dynamic, as providers’ beliefs, motivations, and perceptions are influenced by ongoing changes in the health-care organization, the complexity of which is widely recognized [7].

Most AF interventions that use written or graphical feedback, to our knowledge, provide feedback in the same presentation format for all recipients. In this way, AF is not sensitive to individual differences in barriers to behavior change. This insensitivity could represent a significant limitation for AF, according to psychological theories which suggest that feedback interventions can provoke unintended negative reactions [8,9].

The purpose of this article is to invite debate and direct research attention towards a novel AF component that could guide supervisors in adapting feedback messages for individual health-care providers’ barriers to behavior change: computer-supported feedback message tailoring. We argue that, by leveraging available clinical data, theory-informed knowledge about behavior change, and the knowledge of supervisors or peers who deliver feedback messages, computer-supported tailoring could improve the relevance of feedback messages for behavior change barriers, thereby increasing the effectiveness of AF.

Related work in the field of biomedical informatics offers methods that, to our knowledge, have not been applied for the purpose of tailoring feedback messages for AF. Mitigating the complexity of the environment and clinical cognition to generate relevant feedback messages is a primary goal of medical knowledge-based systems that have been developed and refined over the last half-century [10]. To this end, developers of clinical decision support systems [11], intelligent tutoring systems [12], and computer-interpretable clinical guidelines [13] use knowledge representation methods to provide computer-generated feedback.

Evidence about the effectiveness of message tailoring from the field of computer-tailored health communication (CTHC) suggests that message tailoring may significantly influence health-care provider behavior. Meta-analyses show that CTHC interventions significantly improve health-related behaviors compared to no intervention or a generic message targeting physical exercise, nutritional intake, and chronic disease prevention-associated behaviors [14-16]. CTHC interventions have used psychological theory to guide adaptation of feedback messages over time for individuals [17].

The term “tailoring” is used variably in the literature to describe adapting a behavior change intervention for an individual or population [18]. In the implementation science literature, “tailoring” refers to a process of mapping an intervention to barriers and facilitators of knowledge use in a population [19,20]. We use the concept of “computer-supported tailoring” in an implementation science sense, to mean computer-assisted planning and mapping of an intervention in a dynamic and continuous process that addresses shared and individual barriers and facilitators for the duration of the intervention.

We build our argument on the following assumptions: First, we assume that performance feedback is given routinely to health-care providers for the purpose of knowledge translation, including quality improvement and the implementation of evidence-based practice. Second, we assume that supervisors have some awareness of individual health-care providers’ barriers to change. For example, a supervisor may believe that an individual’s low performance is caused by a lack of motivation rather than lack of knowledge or skill. Third, we assume that supervisors heuristically or intuitively tailor verbal messages when delivering feedback in person. For example, a supervisor may use the “feedback sandwich” technique by “sandwiching” negative feedback between two positive feedback messages [21]. Finally, we assume that the quality of performance data in some cases is adequate to convey meaningful feedback to health-care providers. This last assumption focuses our approach on the selection and visualization of feedback messages, rather than the audit of clinical performance [22]. Therefore, our argument does not speak directly to the improvement of performance measurement within AF.

It is under these conditions, when supervisors interpret credible performance data based in part on their beliefs about individual health-care providers’ barriers to behavior change, that we envision computer-supported feedback message tailoring to have a significant and positive influence. We propose that a software-based feedback tailoring system could guide supervisors efficiently through a message tailoring process to optimize the format of feedback messages for individual health-care providers. We anticipate that such a system, if designed appropriately, could address important problems for supervisors, health-care providers, and AF researchers. For supervisors, a feedback message tailoring system could provide a helpful menu of theory-informed, prioritized feedback messages for each individual recipient. For health-care providers, the system could increase the relevance of feedback and decrease the provision of useless or harmful feedback. For researchers, such a system could enable the observation of tailored AF component effects under heterogeneous and dynamic conditions, to generate knowledge about how and when AF is effective.

The approach we discuss differs from prior work on AF in several ways. First, studies of AF have used tailoring to adapt an intervention to a local context, for example, a country, institution, or a specific clinic. We differentiate the type of tailoring we are describing as being about the design of specific feedback messages, created by a supervisor or peer using a software application, for each individual rather than for a group of providers. Second, the design of feedback messages in AF is typically established prior to the intervention and remains constant, but we propose a mechanism for the continued adaptation of the design prior to the delivery of each message. Third, studies of AF have explored the optimal design of feedback messages, such as comparing the effect of graphical vs textual information or delivery of messages in writing vs in person [23]. This important work, however, has not evaluated messages designed for individual providers rather than for the recipient population. Fourth, many studies of AF use a report that includes a static set of performance measures, such as a graph showing both a process measure of provider’s prescribing behavior and an outcome measure of clinical test results for the provider’s patients. While this kind of report is completely relevant to our discussion, we discuss message tailoring at the level of each performance measure, for example, prioritizing one measure over another, conditional on factors that are most likely to lead to performance improvement rather than sending a static set of indicators to all providers. Finally, we bound the scope of our discussion to address routine, unsolicited feedback, excluding feedback provided outside of the intervention context (e.g., in response to feedback-seeking behavior [24]).

We begin by addressing the significance of data quality and the heterogeneity of AF, using the example of antimicrobial stewardship. Next, we describe the use of psychological theory to inform message tailoring and to support the provision of feedback via a supervisor or peer. We then discuss examples of theoretical constructs that are relevant for feedback message tailoring and provide examples of tailored messages that could be used.

## Discussion

Understanding when AF is effective is important because of its broad use. The potential for AF to improve health care would seem to be increased by unprecedented growth in the adoption of electronic health (eHealth) information technology [25,26], the definition of which can include any use of information and communication technology in the delivery of health care [27]. However, long-standing challenges to using eHealth data for quality improvement and research persist [28], with poor data quality being a central problem [29,30]. Although the routine assessment of data quality is essential for effective secondary use of eHealth data, it is beyond the scope of our discussion. We anticipate that data quality challenges will be gradually reduced as greater quantities of data become available for analysis, as information system designs improve, and with better data analysis techniques [31]. In the remainder of the discussion, we focus on the adaptation of performance feedback occurring after completion of a satisfactory data quality assessment.

AF includes differing components used to target diverse clinical behaviors. Behavior-related diversity includes categories of routineness, disease-focus, and medical specialization. AF has been used to target routine behaviors such as hand hygiene, test ordering, screening, and referral that are relevant across medical domains. AF also targets related groups of behaviors associated with the management of a particular disease, such as the management of diabetes and ischemic heart disease [32]. Unlike routine behaviors and disease-focused behavior groups, AF has been used to target improvement of specialized clinical skills like ultrasonography [33], surgical technique [34], and diagnostic mammography [35]. Within a single category of a targeted behavior, intervention components are heterogeneous with regard to approaches to providing feedback, professional roles of targeted providers, and influence on barriers to behavior change.

We illustrate the heterogeneity of AF using the example domain of antimicrobial stewardship. Antimicrobial stewardship features a variety of behaviors, barriers to change, performance measures, AF components, clinical settings, and professional roles that is representative of AF in general (see Additional file 1). This diversity creates complexity for tailoring feedback messages to influence individual’s barriers to behavior change, given the individual and situational contingencies that create a high-dimensional problem space. Understanding the causal mechanisms that AF components operate upon to influence barriers within this problem space is a challenge for which psychological theory offers many potential solutions.

### Using theory to tailor feedback messages

We view the systematic use of theory as efficacious for understanding causal relationships between elements of AF. We recognize the validity of pragmatic and empiric approaches to conducting research that have been debated [36,37]. Psychological theory offers many credible explanatory mechanisms that could be used to understand how to improve AF [38-40], but AF research has rarely explicitly used theory to inform intervention design, and no consensus has been established for a theoretical approach to AF research [41].

Multiple theories offer one or more causal mechanisms that could motivate and guide feedback message tailoring. Because of the diversity of approaches, contexts, and barriers to behavior change in AF, we anticipate that no single theory encompasses all of the causal mechanisms that might be used to improve performance feedback in a specific setting. For this reason, we use a “menu of constructs” approach, which involves the selection and evaluation of theoretical constructs from many relevant theories to create new representations of a network of causal mechanisms that may mediate the effects of a behavior change intervention [1]. The set of constructs we selected are intended to sufficiently support our argument, but not to definitively survey the theoretical landscape. We apply the menu of constructs approach using two frameworks designed to guide behavior change interventionists in the use of theory. These are the Theoretical Domains Framework (TDF) [40] and the capability, opportunity, motivation, and behavior (COM-B) framework for understanding behavior [42].

**Figure 1:**
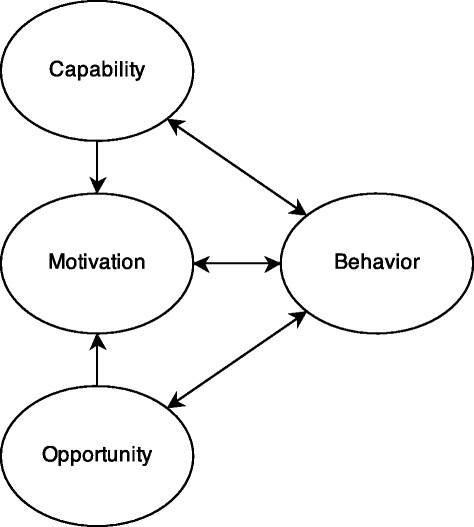
**The capability, opportunity, motivation, and behavior (COM-B) framework for understanding behavior [** 42 **].**

To guide feedback tailoring using theoretical constructs, we have selected examples of constructs from the TDF [40]. The TDF is a taxonomy of 13 behavior change theory categories that researchers can use to identify potentially relevant theory for a behavior change intervention. Within each category is a coherent and validated set of theoretical constructs. An example of a TDF domain is “Beliefs about Capabilities” which contains “Self-efficacy”, a construct from social cognitive theory that has been widely studied [43]. Each theoretical construct within the TDF asserts one or more causal mechanisms that are relevant to behavior change interventions. We chose to use the TDF because it is the only framework that, to our knowledge, was developed by expert consensus and can make a claim to comprehensively including all theoretical constructs that are relevant for behavior change interventions. To understand meaningful differences between barriers to behavior change, we use the COM-B framework for understanding behavior (Figure 1) [42]. COM-B models the determinants of behavior, all of which correspond with specific barriers or facilitators of behavior change: *Capability* refers to determinants such as an individual’s knowledge, skills, and beliefs that create the capacity to engage in a behavior. *Opportunity* contains the environmental influences and other external processes that influence a behavior. An individual’s *motivation* refers to cognitive, emotional, and other psychological processes that direct or stimulate behavior. Behavior influences and is influenced by determinants in the other three categories. For each individual, barriers to behavior change are manifested in one or more COM-B categories.

COM-B categories have been mapped to the TDF domains to guide researchers in selecting the most relevant TDF domain for a specific behavior change intervention [40]. For example, the TDF domain of “Beliefs about Capabilities” was mapped to the COM-B “motivation” category. We chose to use COM-B because it offers intuitive categories of the influences of behavior and because its validated mapping to the TDF provides a useful higher-level categorization of theoretical constructs. Used together, the TDF and COM-B can enable researchers to identify relevant theoretical constructs associated with barriers for a specific behavior that they are aiming to change.

### Understanding barriers to behavior change

To explore the heterogeneity of barriers to behavior change, we discuss examples of antimicrobial stewardship behaviors using COM-B. Each example is informed by a scenario in which a supervisor who is giving verbal feedback to an individual might tailor feedback messages in accordance with changes in the environment or her perceptions of the recipient.

#### Capability barriers

Capability barriers to behavior change are a lack of knowledge or skill that an individual must possess to conduct a behavior. Behaviors targeted by AF commonly require individuals to possess multiple, coordinated capabilities. For example, prescribing requires both medical decision-making and patient communication skills. Differences in provider training, work experience, knowledge maintenance, and innate abilities can contribute to capability differences.

Supervisors may accommodate differences in capability-associated barriers by tailoring feedback messages to address recognizable barriers. For example, reduction of unnecessary antibiotic prescribing requires capabilities in terms of domain knowledge, to recognize the conditions under which prescribing should be delayed, and interpersonal skills to persuade a patient that prescribing antibiotics is not the best action to take. Poor performance in reducing unnecessary antibiotic prescribing could result from lack of either knowledge or skill capability. Consider a supervisor who believes that a low-performing physician has adequate domain knowledge for reducing unnecessary antibiotic prescribing but lacks patient communication skills as evidenced by his patient experience survey scores. To address the most likely capability barrier for the low-performing physician, the supervisor might not focus on the negative performance information but instead reassure the physician about her confidence in his medical knowledge and recommend training to enable the physician to develop better communication skills. For a high-performing physician, giving feedback about antibiotic prescribing would represent a low-priority task because of the physician’s demonstrated competence. As performance improves over time, repeated feedback indicating high performance demonstrates the acquisition of all necessary capabilities, and therefore, it loses priority among feedback messages because of its lower informational value and lower potential to change behavior.

#### Opportunity barriers

Opportunity barriers are external constraints on a provider’s enactment of a behavior. Behavior in clinical settings has multiple, dynamic opportunity barriers. From an informatics perspective, considering the clinical environment to be a complex socio-technical system [44], we provide the following examples of opportunity barriers that are typically not accommodated by AF:

- *Large problem spaces*: For example, clinical guidelines frequently do not address interaction between multiple medical problems within a patient.
- *Disruptions*: Medical emergencies, infrastructure failure, and disease outbreaks are rarely acknowledged by routine audit.
- *Uncertainty*: Patients presenting with multiple symptoms may lead to diagnostic uncertainty not addressed by a guideline.
- *Social influence*: Pressure from patients and co-workers must be negotiated, is dynamic, and can lead to goal conflict.
- *Automation*: Technology can constrain behavior as it becomes embedded in the cognitive work of health care, yet it may also cause unintended errors.

When a supervisor gives face-to-face feedback to a health-care provider, the supervisor can interpret performance reports using a wealth of information from his own experience of events which have occurred during the reporting period. At best, conventional audit measures accurately represent the environment with regard to a narrow set of information that the individual may not be monitoring. However, even in an ideal situation, there is potential for environmental factors to influence behavior in unpredictable ways. In low-resource settings, opportunity barriers may have more significant influences on behavior. For example, a shortage of antibiotic drugs in a low-resource setting creates a barrier that can artificially improve performance of unnecessary prescribing until the drug becomes available.

#### Motivational barriers

Motivational barriers refer to the internal psychological and cognitive processes that prevent individuals from engaging in a behavior. AF addresses behaviors with motivation-associated barriers that are multi-dimensional and can change from situation to situation, such as beliefs, emotions, intentions, goals, and identity [40]. Individual differences in motivation are reflected by variable desire and intent to respond to feedback messages, which can be moderated by perceptions of the feedback messages and feedback source [45].

For example, emotions have a significant role in feedback interventions because individuals perceive performance feedback through their own emotional and reasoning filters [46]. Supervisors who provide performance feedback may aim to emotionally prepare individuals to receive performance feedback. The “feedback sandwich” has been widely used to deliver negative feedback messages, although research suggests that the technique is not effective [21]. Nevertheless, the technique demonstrates that supervisors heuristically tailor feedback messages to accommodate recipient emotions.

The examples of capability, opportunity, and motivational barriers that we have discussed illustrate two potentially significant features of AF that are relevant to feedback tailoring. Firstly, supervisors have some awareness of the nature of a recipient’s specific barriers to behavior change. Secondly, message tailoring of verbal feedback is potentially a significant mediator, associated with the supervisor, that could influence the effectiveness of AF. We propose that a feedback message tailoring system could present a menu of tailored messages for a supervisor to select based on her awareness of the recipient’s specific barriers to behavior change. Such a menu could be created via the repeated interpretation of the individual’s performance data through the lenses of several theoretical constructs that may be salient.

#### Tailoring feedback using a menu of constructs

We use constructs from the TDF to demonstrate how theory may guide a feedback tailoring process. We have selected six examples of constructs, each from a different TDF domain, with two constructs mapped to each of the three COM-B categories (Table 1). We have conducted a preliminary mapping of these constructs to barriers to antibiotic prescribing to demonstrate the relevance of some constructs to hypothetical causal mechanisms that performance feedback may leverage. These six constructs and the causal mechanisms they contain are examples from what we anticipate as a broader set of constructs that could guide message tailoring. Our goal in discussing these examples is to describe a range of constructs and their implications for tailoring. We aim to show how feedback message elements might differentially impact behavior according to specific barriers to behavior change. We anticipate that this “menu of constructs” approach could facilitate the discovery of differences in the salience of constructs across different clinical settings.

**Table 1:** **Preliminary mapping of behavior change barriers for antibiotic prescribing to theoretical constructs**

**COM-B categories [** 42 **]**	**TDF domain [** 40 **]**	**TDF construct [** 40 **]**	**Barrier to antibiotic prescribing [** 47 **]**	**Hypothetical causal mechanism**
Capability	Knowledge	Knowledge of condition/ scientific rationale	Lack of knowledge and training	Feedback can change awareness to impart new knowledge that leads to behavior change
	Skills	Interpersonal skills	Perception of patient demands and preferences	None (feedback has no direct influence on interpersonal skills)
Opportunity	Environmental context and resources	Material resources	Inadequate drug supply infrastructure	None (feedback has no direct influence on material resources)
	Social influences	Social pressure	Peer pressure and social norms	None (feedback has no direct influence on social pressure)
Motivation	Beliefs about capabilities	Self-efficacy	None (barriers are indirect via beliefs about capability constructs)	Feedback can influence perceptions of ability, improving or worsening self-efficacy, which can lead to behavior change
	Emotion	Fear	Fear of bad clinical outcomes	Feedback can cause emotional reactions that influence motivation, leading to behavior change

*Knowledge (including knowledge of condition/scientific rationale)* is a construct from the “Knowledge” TDF domain, defined as “an awareness of the existence of something” [40]. “Knowledge of a condition or scientific rationale for a behavior” as a barrier to behavior change can be directly impacted by a feedback message when the recipient lacks the targeted knowledge. Feedback will be less relevant when provided to an individual who already has the knowledge required to conduct a behavior. For example, to improve unnecessary antibiotic prescribing, providers must know the specific clinical conditions under which prescribing can be avoided. Providers who already know the conditions will find feedback about performance less relevant with regard to this narrow dimension of the prescribing behavior.

*Interpersonal skills* are a construct in the “Skills” TDF domain, defined as “an aptitude enabling a person to carry on effective relationships with others, such as an ability to cooperate, to assume appropriate social responsibilities, or to exhibit adequate flexibility” [40]. Interpersonal skills are important as a capability barrier that, if salient, are unlikely to be directly affected by performance feedback. In the case of antibiotic prescribing, where perceived patient demand is a barrier to behavior change, poor interpersonal skills may cause a provider to acquiesce to a patient’s request for antibiotics if the provider feels ill-equipped to deny the patient a prescription at the risk of damaging the patient-provider relationship. In this case, training is more likely to lead to improved provider capability that enables behavior change, whereas repeated negative feedback about poor performance could potentially reinforce a provider’s beliefs about a lack of interpersonal skills, worsening future performance.

*Material resources* are a construct in the “Environmental Context and Resources” domain of the TDF, defined as “commodities and human resources used in enacting a behavior” [40]. Material resources are associated with feedback in that recipients who lack resources necessary to enact a behavior are likely to find performance feedback less relevant, whereas recipients with adequate resources are likely to find feedback to be more relevant. Feedback that targets resource stewardship can be confounded by resource limitations that artificially improve performance. In situations like a shortage of antibiotic drugs, feedback about unnecessary prescribing is less relevant to the decision to prescribe an antibiotic.

*Social pressure* is a construct in the “Social influences” domain of the TDF. Social pressure is defined as “the exertion of influence on a person or group by another person or group” [40]. The construct of social pressure is important for feedback effectiveness as a situational characteristic that could indicate when peer comparison feedback should be used. As group performance changes from low to high, the presence of social pressure, if salient, could be presumed to influence individuals to move towards the group performance mean. When group performance is low, peer comparison feedback showing a peer-based, upper percentile benchmark may be undermined by individuals’ awareness of the performance of the group. To improve the effect of feedback on performance, social pressure could be accommodated by withholding comparative feedback until a significant percentage of the group had achieved a high level of performance.

*Self-efficacy* is a construct in the “Beliefs about Capabilities” domain of the TDF. Self-efficacy is an individual’s perceived ability to control his own performance and the events that affect him, using the resources at hand [48,49]. In cases where self-efficacy for a given task is low, repeated negative feedback or peer comparison feedback showing diminishing performance relative to peers may worsen the recipient’s self-efficacy [50]. This could lead an individual more quickly towards goal abandonment rather than increased effort to improve performance. Perceptions of patient preferences and demands could represent a formidable barrier to improving performance for a physician who prescribes antibiotics unnecessarily because of poor interpersonal skills. If the physician does not improve, repeatedly showing poor or declining performance scores could lower the physician’s self-efficacy for unnecessary antibiotic prescribing, motivating avoidance behaviors rather than improved performance. A more appropriate solution could be to emphasize relative improvement where it exists. Another potential solution would be to withhold repeated negative feedback and instead offer the low-performing physician training that could lead to performance improvement.

*Fear* is defined as “an intense emotion aroused by the detection of imminent threat, involving an immediate alarm reaction that mobilizes the organism by triggering a set of physiological changes” [40]. Fear is a construct in the “Emotion” domain of the TDF. When the construct of fear is salient, the recipient’s emotional state may interfere with the perception of feedback messages, diminishing the effectiveness of the intervention. For example, if a provider fears that withholding a prescription will worsen outcomes, feedback showing poor outcomes could trigger a physiological response that causes the recipient to reject information provided on the report. In this case, feedback could have high personal relevance for the provider but not be effective for improving performance.

The six constructs we discuss span all three COM-B categories and six of the 13 TDF domains. We anticipate that these constructs are a small proportion of the set of constructs that can be used to tailor feedback messages. A feedback message tailoring system could use the above constructs to guide a supervisor in tailoring feedback for many possible barriers. In the following section, to illustrate how these constructs could be operationalized in an AF intervention, we describe a scenario in which a supervisor is preparing to give feedback to a low-performing physician.

#### Scenario: high occurrence of unnecessary antibiotic prescribing for acute respiratory infection

Dr. A is a supervising physician who is responsible for implementing an antimicrobial stewardship program in her hospital department. Dr. A measures individual prescribing of antibiotics for patients diagnosed with acute respiratory infection (ARI) for each provider in her department. She uses an inverse proportional measure of prescribing behavior (0% is completely compliant) that is calculated as follows:

*Numerator:* Number of patients diagnosed with ARI for whom antibiotics are not indicated AND antibiotics were prescribed.

*Denominator:* Number of patients diagnosed with ARI for whom antibiotics are not indicated.

Dr. A also calculates an average score for the top 10% of providers, to create an achievable performance benchmark against which providers can be compared.

Dr. B is a physician in Dr. A’s department who has performed consistently poor, relative to his peers, over the previous year. Dr. A is preparing performance feedback to review with Dr. B, whose performance data is shown in Table 2. Dr. A intends to discuss the data in Table 2 with Dr. B in a manner that is most likely to lead to performance improvement.

**Table 2:** **Unnecessary antibiotic prescribing performance for an individual provider compared with an achievable peer benchmark**

**Quarter**	**Individual performance (%)**	**Top 10% benchmark (%)**
2013 Q3	86.5	47.5
2013 Q4	84.3	46.2
2014 Q1	85.9	41.3
2014 Q2	80.1	38.8

In this scenario, we assume that individualized performance feedback about antibiotic prescribing behavior for ARI can and should be provided for the following reasons:

1. Performance barriers for the behavior are associated with individual physicians (e.g., capability and motivation) rather than situational constraints, and Dr. A is not aware of any disruptions during the reporting period that would have significantly influenced performance for this measure.
2. Antibiotic prescribing behavior for ARI is not a team-associated behavior (e.g., does not require significant task coordination across providers); therefore, individual performance feedback for this behavior is more relevant than group feedback.
3. Dr. A has assessed the quality of the clinical data and found it to be acceptable.
4. Behavior change for this measure is evidence-based and achievable and is therefore a priority.

**Figure 2:**
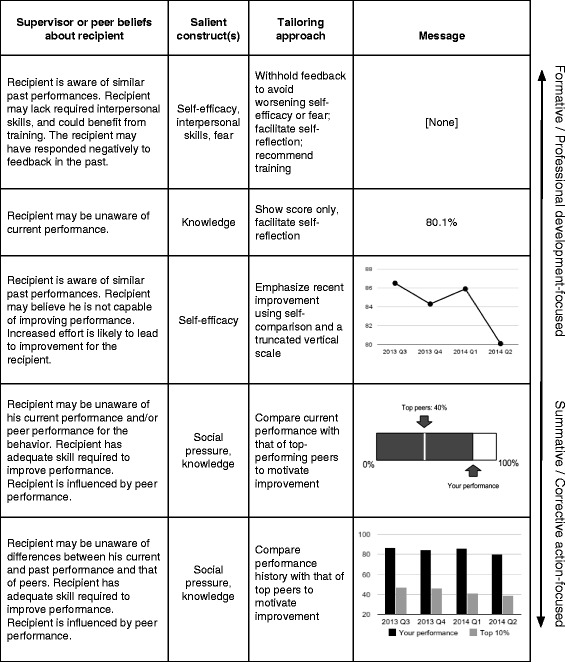
**A prototype menu of feedback messages to support provision of individually tailored feedback.** The feedback messages in the menu are tailored versions of the same performance data from Table 2 based on an inverse performance measure in which 0% indicates the best possible performance for unnecessary antibiotic prescribing. To use the menu, a supervisor could find a description in the leftmost column that most closely matches her own beliefs about the recipient to identify a theory-informed, tailored feedback message in the rightmost column that targets the recipient’s specific barriers to behavior change.

A feedback message tailoring system could analyze the performance data in Table 2 and knowledge about antibiotic prescribing behavior to create a menu of graphical and textual messages (Figure 2). To use the menu, a supervisor like Dr. A could review and select one or more items according to her perceptions of Dr. B’s specific barriers to behavior change. Figure 2 contains three graphical messages that are illustrative examples, and each is based on published studies of AF: scale truncation [35], peer benchmarking [51], and historical peer comparison [52].

### Computer-supported feedback message tailoring

We propose that computer-supported feedback message tailoring could improve the relevance of feedback messages and thereby the effectiveness of AF. This approach focuses on the tailored presentation of performance data, rather than the audit of clinical performance. We focus on this side of AF for two reasons: firstly because this approach enables opportunistic provision of feedback, accommodating increasing availability of performance data when it is of adequate quality. Secondly, we anticipate that this approach will improve understanding of when and how to give feedback, which in turn can inform the optimization of efforts to improve clinical audit.

The examples of tailored feedback in Figure 2 could be generated by a software algorithm that identifies features of an individual provider’s performance and then creates the menu of possible messages that a supervisor could select. To determine how to tailor a message for a recipient, the system could use facts about the provider’s performance history, theoretical constructs, and the clinical context. For example, to generate the graph in Figure 2 featuring a truncated scale, the system would need to “know” the following possible facts:

1. Low performance for unnecessary antibiotic prescribing in this setting is any value above 70%
2. Repeated low performance may be associated with low self-efficacy
3. Self-efficacy can be positively influenced by feedback messages that emphasize improvement
4. Truncated scale graphs can be used to emphasize improvement
5. Truncated scale graphs can be created when current performance shows an improvement of more than 5% over previous performance

Creating a knowledge-base that contains facts like those above is an initial step towards the development of a feedback message tailoring system. Key challenges for creating a feedback tailoring knowledge-base are to develop a valid classification of feedback message elements and to establish performance thresholds that match the expectations of health-care providers. Defining and representing these elements will involve the development of other novel forms of knowledge representation for AF. For example, we do not know the set of message tailoring actions (e.g., graphical scale truncation, withholding, prioritizing, message-based psychological priming) that are meaningful for AF. Moreover, we do not know if different visualizations of performance feedback like those displayed in Figure 2 moderate the effect of feedback on performance.

These areas of uncertainty represent important challenges given the increasing availability of clinical data that enables performance measurement. Much of this work could build directly upon ongoing efforts to formalize terminology for intervention specification and reporting [53,54], and frameworks that facilitate the systematic use of theory, like the TDF and COM-B. We view the formalization of theory-informed implementation knowledge as a foundation for the development of feedback message tailoring knowledge-bases.

The computer-supported feedback message tailoring approach that we describe will likely complement related interventions to change provider behavior in clinical settings. This approach could be characterized as supporting academic detailing (also known as educational outreach visits), an intervention which frequently includes the provision of feedback. Evidence from the most recent Cochrane review of AF indicates that the combined use of academic detailing with AF is generally more effective that using AF alone [2]. Computer-supported feedback message tailoring may enhance the work of academic detailing by providing an automatically generated menu of theory-informed messages that could be selected, saving the time that would be required to manually prepare such a message. In contrast to academic detailing, we envision computer-supported feedback message tailoring as a broader intervention to be used by supervisors without specialized training in educational approaches to behavior change. A primary difference between the intervention we describe and academic detailing is the aim of understanding when feedback may or may not be helpful for overcoming capability and motivational barriers to behavior change. An important part of this vision is the creation of a mechanism that aids supervisors in understanding when feedback is likely to be unhelpful or even detrimental, to avoid negatively impacting performance via the provision of performance feedback.

Computer-supported feedback message tailoring may also resemble techniques used by pharmaceutical sales representatives who collect and analyze physician characteristics and behavioral data to individually tailor persuasive interventions that target prescribing behavior [55,56]. Research about individually tailoring marketing communication for physicians suggests that marketing techniques could also provide helpful models for computer-supported feedback message tailoring [57].

Another body of indirectly related research that informs the approach we propose concerns research about adaptive automated feedback within intelligent tutoring systems and computer-supported cooperative work. Studies of computer-supported argumentation systems [58] and cognitive tutors that facilitate the development of communication skills [59,60] have observed the effects of adaptive feedback on communication behavior. Related work has explored the effects of contrasting visualizations of feedback on communication behavior [61], developed selection strategies for adaptive feedback [62], and evaluated automated, peer-moderated adaptive feedback about intercultural communication skills [63]. This research contributes knowledge about information system design and demonstrates the use of tailored feedback to support learning and behavior change.

## Summary

AF can significantly impact the implementation of evidence-based practice. However, extensive research has not answered questions of how and when AF works [2]. In response to a call for new approaches to AF research [3], we argue that AF research should address a promising and novel AF component: computer-supported feedback message tailoring. The reasons that this approach deserves further attention are many: The potential significance of the systems we envision is growing with our increasing understanding of how to use eHealth data for comparative effectiveness research [30]. The development of standardized terminologies [53,54,64] and common theoretical frameworks [40,42] are creating a basis for the use and operationalization of computer-interpretable implementation knowledge. Evidence about the use of computer-based message tailoring for health behavior change [14] and a significant understanding of knowledge-based computer systems in biomedical informatics [10] reveal a foundation of knowledge and tools that could support the development of message tailoring systems for AF. Finally, and perhaps most importantly, computer-supported feedback message tailoring could benefit several stakeholder groups, including supervisors who deal with much uncertainty and unanticipated reactions when giving feedback to health-care providers.

### Limitations

Our argument has several limitations. A primary limitation is that our approach is contingent on the ability of a supervisor to accurately perceive specific barriers for each individual. The ability to identify barriers can be expected to vary across supervisors and situations and could contribute to the ineffectiveness of feedback. However, we note that, compared to feedback which is not tailored for specific barriers, such a tool might provide relative improvement for the effect of feedback messages. Nevertheless, we do not know the extent to which potential negative effects of making inaccurate assumptions about barriers to behavior change could negatively impact performance. Furthermore, while CTHC evidence suggests that message tailoring can significantly impact health-related behavior, we lack evidence about the comparative effect of visualizations of performance data on clinical behavior. We acknowledge that this discussion is not based in empirical observation and rests on several stated assumptions that do not apply to all AF contexts. Therefore, our argument is subject to biases such as techno-enthusiasm, which we have sought to minimize.

We believe that discussion of innovative approaches like computer-supported feedback message tailoring is needed to spur research interest that can lead to success in understanding when and how AF is effective. We have argued that computer-supported feedback tailoring holds significant potential for the improvement of AF. In pursuing the goal of understanding how to develop feedback message tailoring tools, we are developing a prototype system that we plan to evaluate in disparate AF settings. This work is perhaps best characterized as embracing the complexity of health care by developing adaptive tools to target individual providers’ specific barriers to the adoption of evidence-based practice.

## Additional file

Additional file 1
**Audit and feedback example: promoting antimicrobial stewardship.** The file describes antimicrobial stewardship programs, behavior change barriers for antimicrobial stewardship, and the range of AF interventions that have been used to support the implementation of these programs.

## Abbreviations

AF: Audit and feedback
CTHC: Computer-tailored health communication
COM-B: Capability, Opportunity, Motivation, and Behavior framework
eHealth: Electronic health information technology
TDF: Theoretical Domains Framework

## References

[1] Brehaut JC, Eva KW (2012). Building theories of knowledge translation interventions: use the entire menu of constructs. Implementation Sci.

[2] Ivers N, Jamtvedt G, Flottorp S, Young JM, Odgaard-Jensen J, French SD (2012). Audit and feedback: effects on professional practice and healthcare outcomes. Cochrane Database Syst Rev. (Online).

[3] Ivers NM, Sales A, Colquhoun H, Michie S, Foy R, Francis JJ (2014). No more ‘business as usual’ with audit and feedback interventions: towards an agenda for a reinvigorated intervention. Implementation Sci.

[4] Grol R, Wensing M (2004). What drives change? Barriers to and incentives for achieving evidence-based practice. Med J Aust.

[5] Fretheim A, Oxman AD, Flottorp S (2004). Improving prescribing of antihypertensive and cholesterol-lowering drugs: a method for identifying and addressing barriers to change. BMC Health Serv Res.

[6] Grol R, Wensing M, Eccles M, Davis D (2013). Improving patient care: the implementation of change in health care.

[7] Plsek PE, Greenhalgh T (2001). The challenge of complexity in health care. BMJ : Br Med J.

[8] Ilgen D, Davis C (2000). Bearing bad news: reactions to negative performance feedback. Appl Psychol.

[9] Kluger AN, Van Dijk D (2010). Feedback, the various tasks of the doctor, and the feedforward alternative. Med Educ.

[10] Biomedical Informatics. Computer applications in health care and biomedicine, 3rd ed. New York: Springer; 2006.

[11] Garg AX, Adhikari NKJ, McDonald H, Rosas-Arellano MP, Devereaux PJ, Beyene J (2005). Effects of computerized clinical decision support systems on practitioner performance and patient outcomes: a systematic review. JAMA : J Am Med Assoc.

[12] Crowley RS, Medvedeva O (2006). An intelligent tutoring system for visual classification problem solving. Artif Intell Med.

[13] Peleg M (2013). Computer-interpretable clinical guidelines: a methodological review. J Biomed Inform.

[14] Krebs P, Prochaska JO, Rossi JS (2010). Defining what works in tailoring: a meta-analysis of computer-tailored interventions for health behavior change. Prev Med.

[15] Portnoy DB, Scott-Sheldon LAJ, Johnson BT, Carey MP (2008). Computer-delivered interventions for health promotion and behavioral risk reduction: a meta-analysis of 75 randomized controlled trials 1988–2007. Prev Med.

[16] Lustria MLA, Noar SM, Cortese J, Van Stee SK, Glueckauf RL, Lee J (2013). A meta-analysis of web-delivered tailored health behavior change interventions. J Health Commun.

[17] Park EJ, McDaniel A, Jung MS (2009). Computerized tailoring of health information. Comput Inf Nursing: CIN.

[18] Kreuter MW, Skinner CS (2000). Tailoring: what’s in a name?. Health Educ Res.

[19] Baker R, Camosso-Stefinovic J, Gillies C, Shaw EJ, Cheater F, Flottorp S (2010). Tailored interventions to overcome identified barriers to change: effects on professional practice and health care outcomes. Cochrane Database Syst Rev.

[20] Heather C, Grimshaw J, Wensing M. Knowledge translation in health care: moving from evidence to practice In: Straus S, Tetroe J, Graham ID, editors. 2nd ed. Oxford: John Wiley & Sons: 2013.

[21] Parkes J, Abercrombie S, McCarty T (2013). Feedback sandwiches affect perceptions but not performance. Adv Health Sci Educ: Theory Pract.

[22] Landis Lewis Z, Mello-Thoms C, Gadabu OJ, Gillespie EM, Douglas GP, Crowley RS (2011). The feasibility of automating audit and feedback for ART guideline adherence in Malawi. J Am Med Inform Assoc.

[23] Hysong SJ (2009). Meta-analysis: audit and feedback features impact effectiveness on care quality. Med Care.

[24] Anseel F, Beatty AS, Shen W, Lievens F, Sackett PR (2015). How are we doing after 30 years? A meta-analytic review of the antecedents and outcomes of feedback-seeking behavior. J Manag.

[25] Black AD, Car J, Pagliari C, Anandan C, Cresswell K, Bokun T (2011). The impact of eHealth on the quality and safety of health care: a systematic overview. PLoS Med.

[26] Wright A, Henkin S, Feblowitz J, McCoy AB, Bates DW, Sittig DF (2013). Early results of the meaningful use program for electronic health records. N Engl J Med.

[27] Oh H, Rizo C, Enkin M, Jadad A (2005). What is eHealth 3: a systematic review of published definitions. J Med Internet Res.

[28] Weiner MG, Embi PJ (2009). Toward reuse of clinical data for research and quality improvement: the end of the beginning?. Ann Intern Med.

[29] Powell A, Davies H, Thomson R (2003). Using routine comparative data to assess the quality of health care: understanding and avoiding common pitfalls. Qual Safety Health Care.

[30] Hersh WR, Weiner MG, Embi PJ, Logan JR, Payne PRO, Bernstam EV (2013). Caveats for the use of operational electronic health record data in comparative effectiveness research. Med Care.

[31] Hripcsak G, Albers DJ (2013). Next-generation phenotyping of electronic health records. J Am Med Inform Assoc.

[32] Ivers NM, Tu K, Young J, Francis JJ, Barnsley J, Shah BR (2013). Feedback GAP: pragmatic, cluster-randomized trial of goal setting and action plans to increase the effectiveness of audit and feedback interventions in primary care. Implementation Sci: IS.

[33] Chalouhi GE, Salomon LJ, Fontanges M, Althuser M, Haddad G, Scemama O (2013). Formative assessment based on an audit and feedback improves nuchal translucency ultrasound image quality. J Ultrasound Med: Official J Am Institute Ultrasound Med.

[34] Simunovic M, Coates A, Smith A, Thabane L, Goldsmith CH, Levine MN (2013). Uptake of an innovation in surgery: observations from the cluster-randomized quality initiative in rectal cancer trial. Can J Surg J Can de chirurgie.

[35] Geller BM, Ichikawa L, Miglioretti DL, Eastman D (2012). Web-based mammography audit feedback. AJR Am J Roentgenol.

[36] Oxman AD, Fretheim A, Flottorp S (2005). The OFF theory of research utilization. J Clin Epidemiol.

[37] Eccles M, Grimshaw J, Walker A, Johnston M, Pitts N (2005). Changing the behavior of healthcare professionals: the use of theory in promoting the uptake of research findings. J Clin Epidemiol.

[38] Grol RPTM, Bosch MC, Hulscher MEJL, Eccles MP, Wensing M (2007). Planning and studying improvement in patient care: the use of theoretical perspectives. Milbank Q.

[39] Michie S, Johnston M, Abraham C, Lawton R, Parker D, Walker A (2005). Making psychological theory useful for implementing evidence based practice: a consensus approach. Qual Safety Health Care.

[40] Cane J, O’Connor D, Michie S (2012). Validation of the theoretical domains framework for use in behaviour change and implementation research. Implementation Sci.

[41] Colquhoun HL, Brehaut JC, Sales A, Ivers N, Grimshaw J, Michie S (2013). A systematic review of the use of theory in randomized controlled trials of audit and feedback. Implementation Sci.

[42] Michie S, Stralen MMv, West R (2011). The behaviour change wheel: a new method for characterising and designing behaviour change interventions. Implementation Sci.

[43] Bandura A (1977). Self-efficacy: Toward a unifying theory of behavioral change. Psychol Rev.

[44] Vicente KJ (1999). Cognitive work analysis: toward safe, productive, and healthy computer-based work.

[45] Ilgen DR, Fisher CD, Taylor MS (1979). Consequences of individual feedback on behavior in organizations. J Appl Psychol.

[46] Eva KW, Armson H, Holmboe E, Lockyer J, Loney E, Mann K (2012). Factors influencing responsiveness to feedback: on the interplay between fear, confidence, and reasoning processes. Adv Health Sci Educ.

[47] WHO (2001). WHO Global Strategy for Containment of Antimicrobial Resistance. Tech. rep.

[48] Silver WS, Mitchell TR, Gist ME (1995). Responses to successful and unsuccessful performance: the moderating effect of self-efficacy on the relationship between performance and attributions. Organ Behav Hum Decis Process.

[49] Bandura A (1993). Perceived self-efficacy in cognitive development and functioning. Educ Psychologist.

[50] Bandura A, Jourden FJ (1991). Self-regulatory mechanisms governing the impact of social comparison on complex decision making. J Pers Soc Psychol.

[51] Kiefe CI, Allison JJ, Williams O, Person SD, Weaver MT, Weissman NW (2001). Improving quality improvement using achievable benchmarks for physician feedback: a randomized controlled trial. JAMA.

[52] Gerber JS, Prasad PA, Fiks AG, Localio AR, Grundmeier RW, Bell LM (2013). Effect of an outpatient antimicrobial stewardship intervention on broad-spectrum antibiotic prescribing by primary care pediatricians: a randomized trial. JAMA.

[53] Colquhoun H, Leeman J, Michie S, Lokker C, Bragge P, Hempel S (2014). Towards a common terminology: a simplified framework of interventions to promote and integrate evidence into health practices, systems, and policies. Implementation Sci.

[54] Waltz TJ, Powell BJ, Chinman MJ, Smith JL, Matthieu MM, Proctor EK (2014). Expert recommendations for implementing change (ERIC): protocol for a mixed methods study. Implementation Sci.

[55] Steinbrook R (2006). For sale: physicians’ prescribing data. N Engl J Med.

[56] Fugh-Berman A, Ahari S (2007). Following the script: how drug reps make friends and influence doctors. PLoS Med.

[57] Narayanan S, Manchanda P, Chintagunta PK (2003). The informative versus persuasive role of marketing communication in new product categories: an application to the prescription antihistamines market. SSRN Scholarly Paper ID 472881.

[58] Scheuer O, Loll F, Pinkwart N, McLaren BM (2010). Computer-supported argumentation: a review of the state of the art. Int J Computer-Supported Collaborative Learn.

[59] Walker E, Ogan A, Aleven V, Jones C. Two approaches for providing adaptive support for discussion in an ill-defined domain. Intelligent Tutoring Systems for Ill-Defined Domains: Assessment and Feedback in Ill-Defined Domains; 2008, p. 1.

[60] Diamant EI, Lim BY, Echenique A, Leshed G, Fussell SR (2009). Supporting intercultural collaboration with dynamic feedback systems: preliminary evidence from a creative design task. CHI ’09 Extended Abstracts on Human Factors in Computing Systems CHI ’09.

[61] Leshed G, Perez D, Hancock JT, Cosley D, Birnholtz J, Lee S (2009). Visualizing real-time language-based feedback on teamwork behavior in computer-mediated groups. Proceedings of the SIGCHI conference on human factors in computing systems CHI ’09.

[62] Gutierrez F, Atkinson J (2011). Adaptive feedback selection for intelligent tutoring systems. Expert Syst Appl.

[63] Ogan A, Walker E, Aleven V, Jones C. Using a peer moderator to support collaborative cultural discussion. In: CATS 2008: Workshop on culturally-aware tutoring systems: 2008. p. 71.

[64] Michie S, Richardson M, Johnston M, Abraham C, Francis J, Hardeman W (2013). The behavior change technique taxonomy (v1) of 93 hierarchically clustered techniques: building an international consensus for the reporting of behavior change interventions. Ann Behav Med.

