# Supplementary material for: Computer-supported feedback message tailoring: theory-informed adaptation of clinical audit and feedback for learning and behavior change
Source: Implement Sci. 2015 Jan 21;10:12. doi: 10.1186/s13012-014-0203-z (PMC4320482; doi:10.1186/s13012-014-0203-z)
Supplement: Additional file 1 — Audit and feedback example: promoting antimicrobial stewardship. The file describes antimicrobial stewardship programs, behavior change barriers for antimicrobial stewardship, and the range of AF interventions that have been used to support the implementation of these programs. [file 13012_2014_203_MOESM1_ESM.pdf]

Audit and feedback example: promoting antimicrobial stewardship

Overuse of antibiotics is associated with the complex phenomenon of antibiotic resistance, which has persisted as a high-priority public health concern. [1,2] Public health organizations promote the use of multi-faceted antimicrobial stewardship programs to improve clinical outcomes, reduce costs, and reduce the spread of antibacterial resistance. [1,3] Antimicrobial stewardship programs target antibiotic prescribing behaviors such as encouraging reduction of inappropriate antibiotic prescribing (e.g. inappropriately using a broad-spectrum antibiotic when a narrow-spectrum antibiotics is indicated) or unnecessary prescribing (e.g. prescribing an antibiotic when none is clinically indicated). A WHO report on containment of antimicrobial resistance [1] identified the following barriers to behavior change for antibiotic stewardship:

- Lack of knowledge and training
- Lack of access to information
- Lack of diagnostic support
- Fear of bad clinical outcomes
- Perception of patient demands and preferences
- Economic incentives
- Peer pressure and social norms
- Factors associated with the prescriber's working environment
- Lack of appropriate legislation or enforcement of legislation
- Inadequate drug supply infrastructure

To overcome behavior change barriers, programs may use restrictive interventions, such as requiring approval for prescribing of certain classes of antibiotics, or persuasive interventions like educational meetings and audit and feedback (AF). Multi-faceted interventions have been shown to be effective for improving antibiotic prescribing in hospital inpatient settings. [4] Public health organizations advocate for the use of AF as a key behavior change intervention to improve antimicrobial stewardship. [1,5] Nevertheless, evidence about the effectiveness of AF as a behavior change intervention to promote antibiotic stewardship is inconclusive in both ambulatory and inpatient clinical settings. Systematic

reviews of interventions to improve antibiotic prescribing behaviors suggest that the success of interventions depends on the specific prescribing behaviors and specific barriers to behavior change in each setting. [4,6] Used alone, AF appears to have only small effects on prescribing behaviors. [6]

AF interventions that target antibiotic prescribing use heterogeneous performance measurement approaches for differing provider roles. Measurement approaches may involve techniques such as retrospective chart audit [7], daily monitoring and documentation of antibiotic prescription records by a clinical pharmacist [8], or electronic prescribing and reporting tools. [9] Performance summaries about antibiotic prescribing may include process and outcome measures of performance. Process measures reflect the provider's intent or actions, such as the proportion of a population of patients for whom the provider prescribed non-indicated antibiotics. Outcome measures reflect the patient's disease state or other results of care that are causally associated with the provider's behavior. For example, a feedback report could include outcome measures showing the proportion of patients having different bacterial outcomes (e.g. eradication, persistence, or super-infection) or clinical outcomes (e.g. cured, improving, no change or worsening). [8]

Provider roles involved in antibiotic prescribing can differ across healthcare profession and specialization. For example, a study targeting antimicrobial stewardship in a teaching hospital in Australia recognized the importance of the roles of nurses, infectious disease specialists, and pharmacists as potential influences on prescribing behavior for junior and senior physicians. [9] In non-academic clinical settings, provider roles may be similarly expected to differ as non-physician clinicians frequently have antibiotic prescribing authority, including nurse practitioners, physician assistants, and midwives.

References

1. WHO: **WHO Global Strategy for Containment of Antimicrobial Resistance**. Tech. rep., World Health Organization, Geneva, Switzerland 2001, [http://www.who.int/csr/resources/publications/drugresist/WHO_CDS_CSR_DRS_2001_2_EN/en/].

2. Goossens H, Ferech M, Vander Stichele R, Elseviers M, ESAC Project Group: **Outpatient antibiotic use in Europe and association with resistance: a cross-national database study**. *Lancet* 2005, **365**(9459):579–587.

3. Charani E, Edwards R, Sevdalis N, Alexandrou B, Sibley E, Mullett D, Franklin BD, Holmes A: **Behavior change strategies to influence antimicrobial prescribing in acute care: a systematic review**. *Clinical infectious diseases: an official publication of the Infectious Diseases Society of America* 2011, **53**(7):651–662.

4. Davey P, Brown E, Charani E, Fenelon L, Gould IM, Holmes A, Ramsay CR, Wiffen PJ, Wilcox M: **Interventions to improve antibiotic prescribing practices for hospital inpatients**. *The Cochrane database of systematic reviews* 2013, **4**:CD003543.

5. CDC: **Core Elements of Hospital Antibiotic Stewardship Programs**. Tech. rep., US Department of Health and Human Services, CDC, Atlanta, GA 2014, [http://www.cdc.gov/getsmart/healthcare/implementation/core-elements.html].

6. Arnold SR, Straus SE: **Interventions to improve antibiotic prescribing practices in ambulatory care**. *The Cochrane database of systematic reviews* 2005, (4):CD003539.

7. Trautner BW, Kelly PA, Petersen N, Hysong S, Kell H, Liao KS, Patterson JE, Naik AD: **A hospital-site controlled intervention using audit and feedback to implement guidelines concerning inappropriate treatment of catheter-associated asymptomatic bacteriuria**. *Implementation science: IS* 2011, **6**:41.

8. Nathwani D, Gray K, Borland H: **Quality indicators for antibiotic control programmes**. *The Journal of hospital infection* 2002, **50**(3):165–169.

9. Baysari MT, Oliver K, Egan B, Li L, Richardson K, Sandaradura I, Westbrook JI, Day RO: **Audit and feedback of antibiotic use: utilising electronic prescription data**. *Applied clinical informatics* 2013, **4**(4):583–595.
